# MTA1 Regulates EMT and BRAF Signaling Networks in Canine Urothelial Carcinoma

**DOI:** 10.3390/ijms27167272

**Published:** 2026-08-14

**Authors:** Gisella Campanelli, Nema Parkhomovsky, Chun Kuen Mak, Ching Yang, Anait S. Levenson

**Affiliations:** 1 Department of Veterinary Biomedical Sciences, Lewyt College of Veterinary Medicine, Long Island University, Brookville, NY 11548, USA; gisella.campanelli@liu.edu (G.C.);; 2 Lewyt College of Veterinary Medicine, Long Island University, Brookville, NY 11548, USA; 3 Department of Veterinary Clinical Sciences, Lewyt College of Veterinary Medicine, Long Island University, Brookville, NY 11548, USA

**Keywords:** canine urothelial carcinoma, transitional cell carcinoma, canine cell lines, canine xenografts, MTA1, BRAF V595E, EMT, therapeutic target

## Abstract

Metastasis-associated protein 1 (MTA1), an oncogenic transcriptional regulator, is overexpressed in canine urothelial carcinoma (UC) and is associated with aggressive clinicopathological features. However, its functional role and molecular mechanisms in canine UC remain poorly understood. Here, we investigated the contribution of MTA1 to epithelial-to-mesenchymal transition (EMT) and its interaction with BRAF signaling. MTA1 silencing in two canine UC cell lines significantly inhibited cell proliferation, cell survival, migration, and xenograft tumor growth. Mechanistically, MTA1 knockdown reduced the expression of MTA2, MTA3, and COX2, while producing unexpected changes in key EMT regulators, including Snail, Slug, and Cyclin D1, suggesting the activation of compensatory signaling pathways. MTA1 silencing also decreased mutant BRAF expression in AxA cells while increasing wild-type BRAF expression in SH cells, indicating context-dependent regulation of BRAF signaling. In AxA cells, reduced AKT phosphorylation following MTA1 knockdown further supports functional crosstalk between the BRAF and MTA1/AKT signaling pathways. Collectively, these findings identify MTA1 as a critical regulator of canine UC progression and reveal complex signaling interactions that support its potential as a therapeutic target for canine UC.

## 1. Introduction

Tumors of the canine urinary tract account for approximately 2% of all canine neoplasms, with urothelial carcinoma (UC), also known as transitional cell carcinoma, representing the most common histologic subtype [1]. Most canine UCs are invasive, high-grade tumors with a strong propensity for local invasion and distant metastasis, contributing to their poor clinical prognosis [2]. Certain breeds, particularly the Scottish Terrier, exhibit a markedly increased genetic predisposition to UC, largely due to the presence of an activating point mutation in the *BRAF* gene (V595E), the canine homolog of the human BRAF V600E mutation frequently observed in melanoma and other malignancies [3,4,5,6]. Although BRAF mutations are uncommon in human bladder cancer, the BRAF V595E mutation is detected in approximately 80% of canine UC cases and results in constitutive activation of the mitogen-activated protein kinase/extracellular signal-regulated kinase (MAPK/ERK) signaling pathway [7]. In addition to MAPK/ERK signaling, dysregulation of EGFR, p53, PI3K/AKT/mTOR, and cyclooxygenase-2 (COX2) pathways has been implicated in canine UC development and progression [8,9,10,11,12,13].

Current therapeutic approaches for canine UC include chemotherapy, conformal or targeted radiation therapy, and molecularly targeted treatments such as COX2-inhibitory nonsteroidal anti-inflammatory drugs (NSAIDs) [14]. Although COX2 inhibitors, administered alone or in combination with chemotherapy, have shown clinical benefit, overall response rates remain suboptimal [15,16,17]. Furthermore, concerns regarding the cardiovascular toxicity associated with long-term COX2 inhibition limit their therapeutic utility [18,19]. Small-molecule tyrosine kinase inhibitors targeting mutant BRAF, including vemurafenib, dabrafenib, and sorafenib, have demonstrated efficacy in human cancers harboring BRAF V600E mutations and are currently under investigation in veterinary oncology. Early studies indicate that these agents effectively suppress MAPK/ERK signaling in canine UC [7]. However, targeted therapies directed against other relevant pathways, such as EGFR signaling, remain largely experimental and are not yet clinically available. Consequently, there remains an urgent need to identify novel molecular drivers of canine UC and develop more effective targeted therapeutic strategies.

Metastasis-associated protein 1 (MTA1), together with MTA2 and MTA3, is a key component of the nucleosome remodeling and deacetylation (NuRD) complex, a multifunctional chromatin-remodeling and transcriptional regulatory complex implicated in cancer progression [20]. Overexpression of MTA1 has been associated with aggressive tumor behavior, metastatic dissemination, and poor clinical outcomes across numerous human malignancies, including bladder cancer [21,22,23,24]. Recently, we demonstrated that elevated MTA1 expression in canine UC tissues correlates with adverse clinicopathological features, including high tumor grade, muscular and vascular invasion, and metastatic disease [25]. Mechanistically, MTA1 functions as an upstream regulator of several oncogenic signaling pathways, including PTEN/PI3K/AKT/mTOR and p53 signaling, thereby promoting tumor growth, survival, and progression [26,27,28,29,30]. These observations suggest that MTA1 represents a promising yet largely unexplored therapeutic target in canine UC and may functionally interact with oncogenic BRAF signaling. Moreover, our previous findings led us to hypothesize that MTA1 may play a critical role in regulating epithelial-to-mesenchymal transition (EMT), a key process underlying canine UC progression and metastasis [25].

Epithelial-to-mesenchymal transition is a fundamental biological process that contributes to tumor invasion, metastatic dissemination, and therapeutic resistance in many cancers, including UC [31,32]. During EMT, epithelial cells lose cell–cell adhesion and acquire mesenchymal characteristics that enhance migratory and invasive capabilities. These phenotypic changes are accompanied by alterations in hallmark molecular markers, including decreased E-cadherin (E-cad) and increased vimentin expression.

Emerging evidence supports a close association between MTA1 expression and EMT activation. In our previous study, we were the first to demonstrate high MTA1 levels accompanied by reduced E-cad expression in primary canine UC tissues. Interestingly, lung metastatic lesions exhibited markedly elevated MTA1 expression but also showed re-expression of E-cad, suggesting a complex and potentially context-dependent relationship between MTA1 and EMT signaling during metastatic progression, whereas E-cad expression remained consistently elevated across all cell lines examined [25]. These findings suggest that MTA1-mediated regulation of EMT in canine UC may differ from classical EMT paradigms and warrants further investigation. Furthermore, although accumulating evidence implicates MTA1 in cancer progression and metastasis, its role in the molecular pathogenesis of canine UC, particularly in the context of BRAF V595E mutation, remains poorly understood.

The availability of cultured canine UC cell lines provides a unique opportunity to explore these mechanisms. Specifically, AxA cells known to harbor the BRAF V595E mutation express the highest levels of MTA1 among three other cells lines with unknown BRAF status [25]. These complementary models enable us to investigate the functional role of MTA1, its potential relationship with BRAF mutation status, and its contribution to EMT-associated signaling in canine UC.

Therefore, in the present study, we investigated whether MTA1 is functionally associated with BRAF V595E signaling and whether it contributes to EMT-mediated tumor progression in canine UC. Using complementary in vitro and in vivo models, we demonstrate that MTA1 silencing significantly reduces UC cell proliferation, migration, and invasion, and suppresses tumor growth in xenograft models. Importantly, in vitro MTA1 knockdown also uncovered unexpected alterations in key signaling pathways, suggesting the engagement of compensatory regulatory networks following MTA1 inhibition. Collectively, these findings identify MTA1 as a critical regulator of canine UC progression and support its potential as a promising molecular target for future interceptive and therapeutic strategies.

## 2. Results

### 2.1. MTA1 Knockdown Reduces Canine UC Cell Proliferation, Survival, and Migration In Vitro

**Figure 1:**
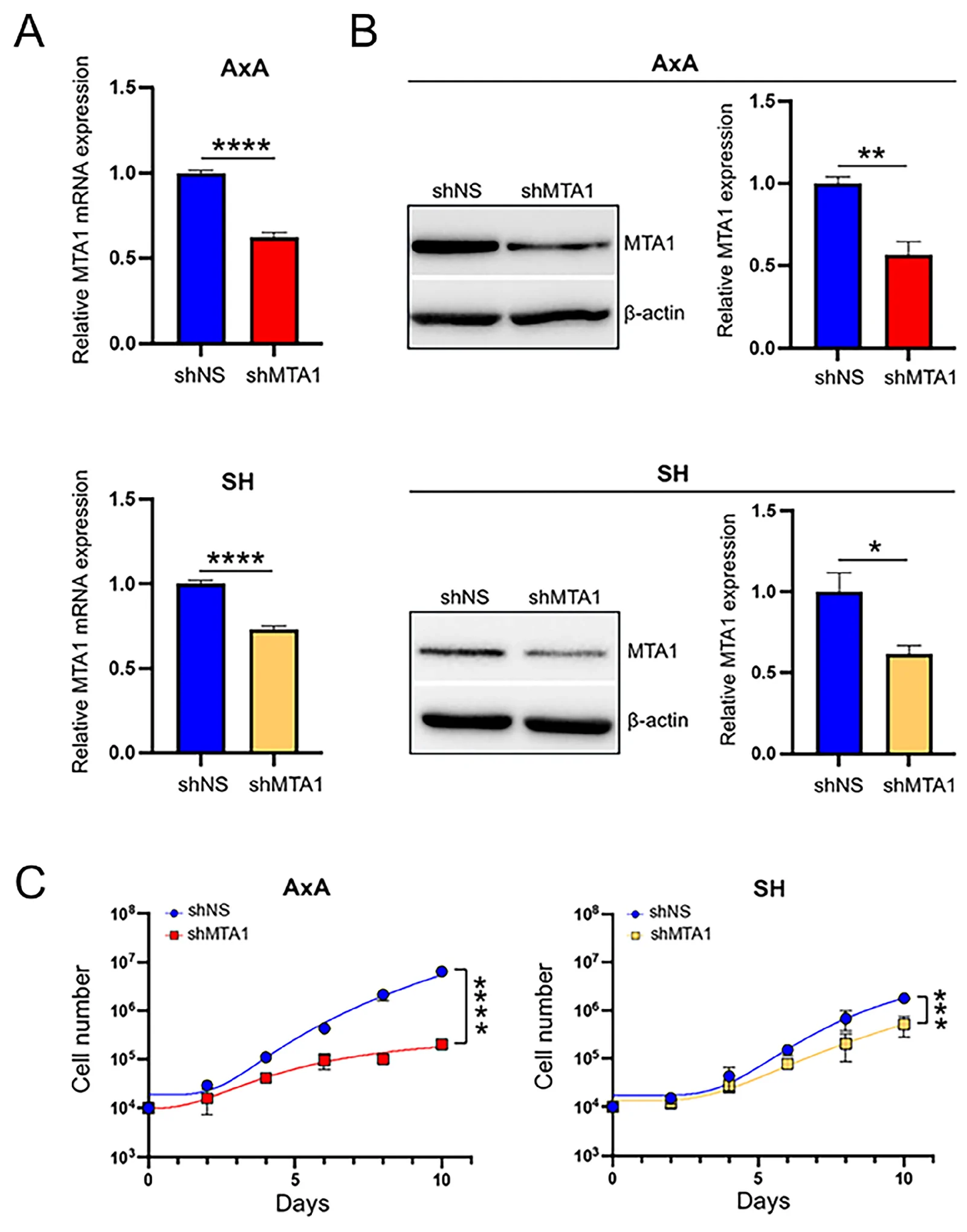
MTA1 knockdown suppresses proliferation of canine UC AxA and SH cells. (**A**) Validation of MTA1 knockdown in AxA cells by qRT-PCR (left) and Western blot analysis (right). β-Actin served as the loading control. (**B**) Validation of MTA1 knockdown in SH cells by qRT-PCR (left) and Western blot analysis (right). β-Actin served as the loading control. MTA1 expression was significantly reduced in shMTA1 cells compared with shNS controls. (**C**) Cell proliferation assays demonstrate reduced growth of AxA-shMTA1 (left) and SH-shMTA1 (right) cells compared with their respective shNS controls. Statistical analysis was performed using *t*-test (AB) and unpaired two-sample *t*-tests for each individual time-point, followed by Bonferroni correction for multiple comparisons. Data are presented as mean ± SD (*n* = 3). * *p* < 0.05; ** *p* < 0.01; *** *p* < 0.001; **** *p* < 0.0001.

We have previously shown that MTA1 is differentially expressed in the four canine UC cell lines: MTA1 protein levels were highest in AxA cells followed by SH cells [25]. To better understand the functional role of MTA1 in canine UC progression, we investigated the consequences of MTA1 silencing in these two cell lines using lentiviral-mediated MTA1-shRNA and control nonspecific shRNA (shNS), described previously [33,34]. MTA1 knockdown efficiency was validated on both mRNA and protein levels (Figure 1A,B). The AxA cell clone with the highest MTA1 expression level achieved a more effective MTA1 knockdown on mRNA (38.0% vs. 26.8%) and more evidently on a protein level (41.3% vs. 38.4%) compared to SH cells. First, the potential role of MTA1 in tumor growth was assessed using a cell proliferation assay. The results indicated that MTA1 knockdown significantly suppressed cell proliferation in both AxA and SH cells (Figure 1C). In agreement with published data [35], AxA control cells had notably shorter population doubling times compared with SH control cells (24.5 ± 0.47 h vs. 27.6 ± 0.52 h). Importantly, MTA1 knockdown cells exhibited a significant increase in doubling time compared to their respective controls in both AxA (65.8 ± 3.64 h) and SH (37.4 ± 0.79 h) cells indicating that MTA1 has a proliferative effect in canine UC cells.

**Figure 2:**
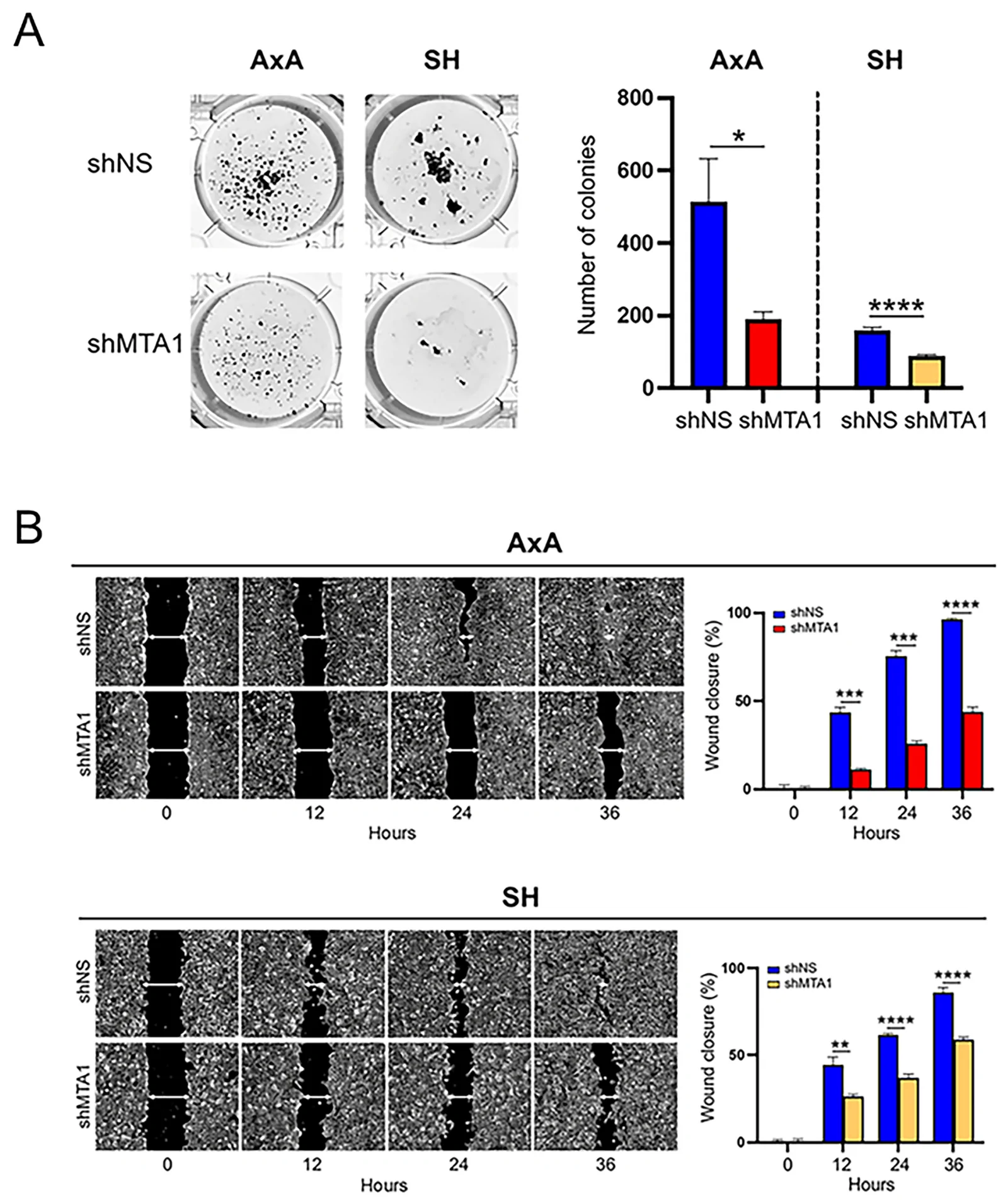
MTA1 knockdown reduces clonogenic survival and migratory capacity of canine UC AxA and SH cells. (**A**) Representative colony formation assays of AxA and SH shNS and shMTA1 cells (left) and corresponding quantification (right), demonstrating significantly reduced clonogenic survival following MTA1 knockdown. (**B**) Representative wound healing assays of AxA-shNS and AxA-shMTA1 cells (upper panel) and SH-shNS and SH-shMTA1 cells (lower panel), showing impaired migratory capacity in MTA1-silenced cells. Statistical analysis was performed using *t*-test (**A**) and mixed-effects model (**B**). Data are presented as mean ± SEM (*n* = 3). * *p* < 0.05; ** *p* < 0.01; *** *p* < 0.001; **** *p* < 0.0001.

Further, we assessed metastasis-associated cellular properties in MTA1 expressing and knockdown cells via colony formation and wound healing assays. As shown in Figure 2A, MTA1 knockdown significantly reduced colony formation in both canine UC cell lines, resulting in fewer and smaller colonies than their respective controls, indicating decreased clonogenic potential. Although both cell lines exhibited reduced colony formation following MTA1 silencing, SH control cells formed fewer but larger colonies than AxA control cells. The inhibitory effect of MTA1 knockdown was more pronounced in AxA cells (55.8% reduction, *p* = 0.0217), whereas SH cells exhibited a more consistent, although less marked, reduction in colony formation (37.1% reduction, *p* < 0.0001). The role of MTA1 in UC cell motility was evaluated using a wound-healing assay. As shown in Figure 2B, MTA1 knockdown significantly impaired migration in both AxA and SH cells, resulting in larger residual wound areas and significantly reduced wound closure compared with their respective control cells expressing MTA1.

Overall, these findings demonstrate that MTA1 promotes canine UC cell proliferation, clonogenic survival, and migration, underscoring its critical role in tumor progression. Notably, MTA1 knockdown produced a greater inhibitory effect on cell proliferation in AxA cells, whereas the reduction in migratory capacity was more pronounced in SH cells. These differential responses highlight the biological heterogeneity of canine UC cells and suggest that SH cells may possess a more migratory or metastatic phenotype than AxA cells.

### 2.2. The Effects of MTA1 Knockdown on EMT-Associated Markers in Canine UC Cells In Vitro

To elucidate the mechanism of action for the oncogenic activity of MTA1 in canine UC, we further examined the molecular consequences of MTA1 knockdown in cells by measuring expression levels of MTA1 family proteins, namely MTA2 and MTA3, and molecular markers associated with EMT events (COX2, E-cad, CK8, vimentin, Snail, Slug, and Cyclin D1).

**Figure 3:**
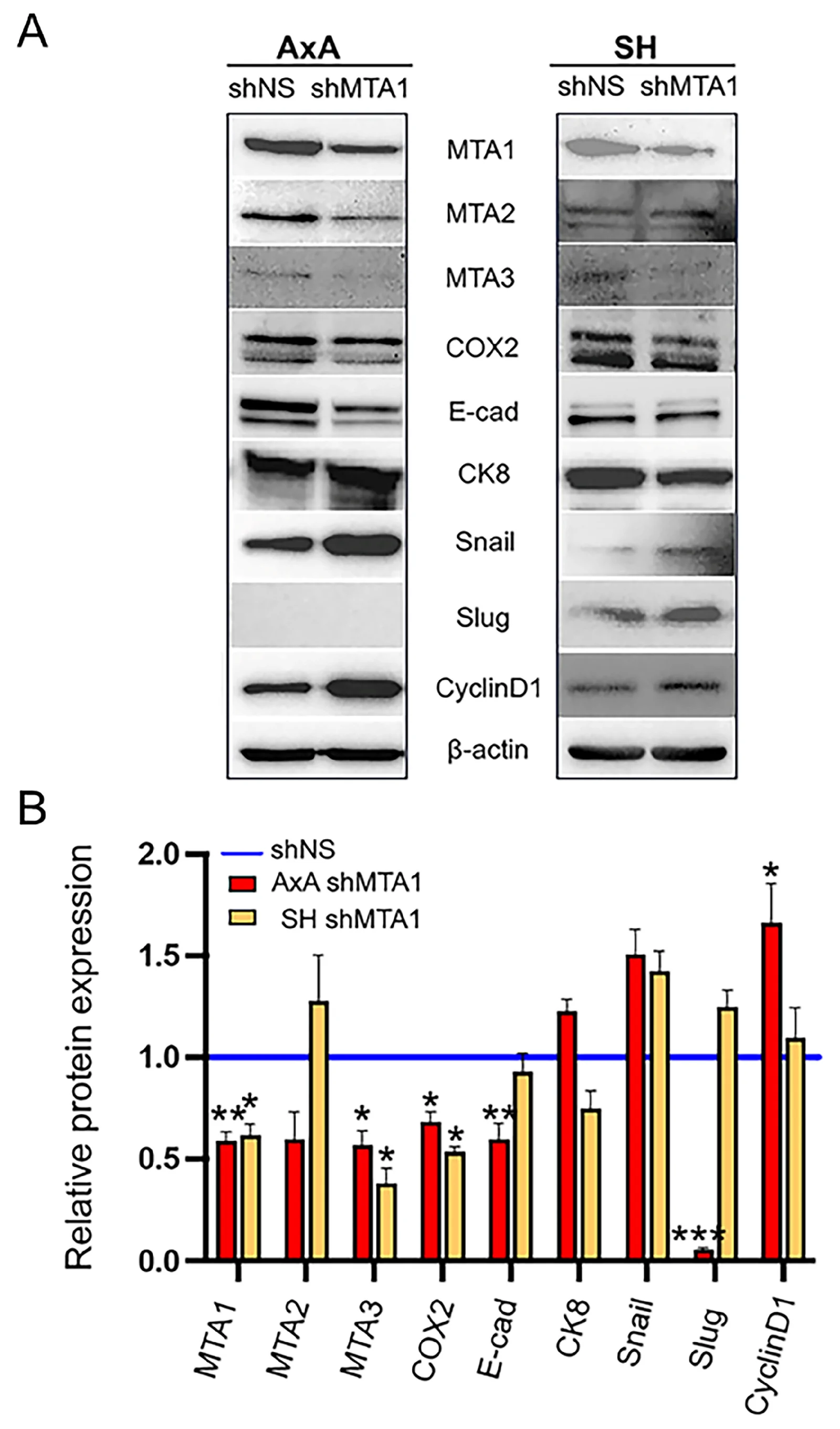
MTA1 knockdown differentially modulates EMT-associated signaling in canine UC AxA and SH cells. (**A**) Representative Western blot analysis of MTA family members and EMT-associated proteins in AxA and SH shNS and shMTA1 cells. β-Actin served as the loading control. (**B**) Densitometric quantification of Western blot data demonstrating differential regulation of EMT-associated markers following MTA1 knockdown, including significant downregulation of MTA3 and COX2 and upregulation of Cyclin D1. Statistical analysis was performed using *t*-test. Data are presented as mean ± SEM (*n* = 4–5). * *p* < 0.05; ** *p* < 0.01; *** *p* < 0.001.

As shown in Figure 3, MTA2 expression was slightly decreased in AxA cells but increased in SH cells following MTA1 silencing, whereas the already low levels of MTA3 were further reduced in both shMTA1 cell lines compared with shNS controls. These findings suggest that MTA1 depletion disrupts the composition and functional integrity of the NuRD complex. Consistent with our previous study, which identified AxA cells as expressing the highest levels of both MTA1 and COX2 among the four canine UC cell lines examined [25], MTA1 knockdown reduced COX2 expression in both AxA and SH cells (Figure 3A,B). In contrast, E-cadherin expression, previously reported to be uniformly high in parental AxA and SH cells [25], exhibited variable changes following MTA1 silencing in AxA cells. MTA1 knockdown also differentially regulated cytokeratin 8 (CK8), increasing its expression in AxA cells while decreasing it in SH cells. Unexpectedly, MTA1 depletion increased the expression of the EMT-associated transcription factors Snail and Slug. In AxA cells, Snail expression was upregulated, whereas Slug remained undetectable. In contrast, SH cells exhibited high basal Slug expression that was further increased following MTA1 knockdown, accompanied by elevated Snail expression. Cyclin D1 levels were also increased in both AxA and SH MTA1 knockdowns (Figure 3A,B). The induction of these pro-proliferative and EMT-associated factors despite MTA1 depletion suggests the activation of compensatory signaling pathways that may partially offset the loss of MTA1-mediated oncogenic functions.

### 2.3. The Effects of MTA1 Knockdown on BRAF-Associated Markers in Canine UC Cells In Vitro

**Figure 4:**
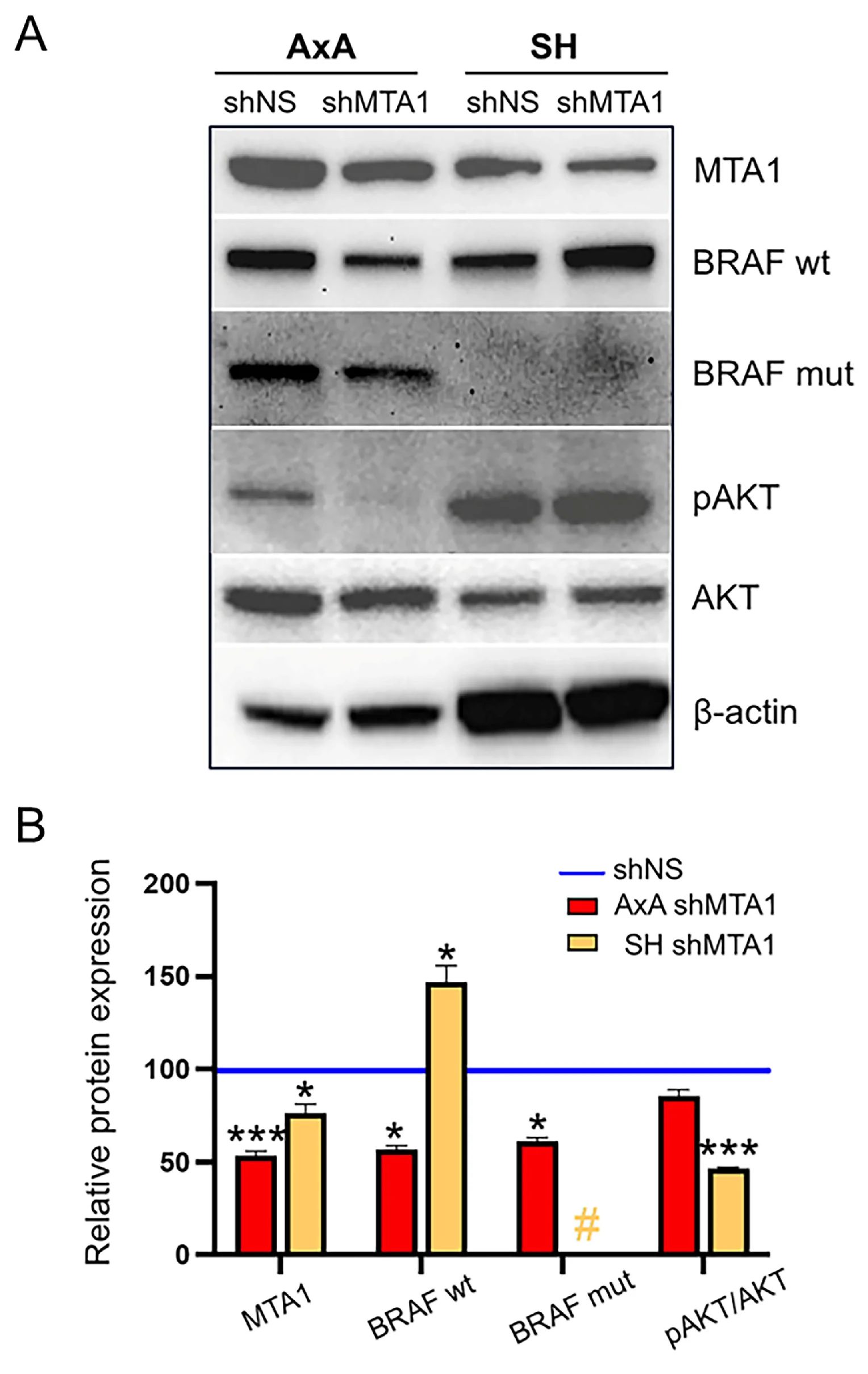
MTA1 knockdown alters BRAF-associated signaling in canine UC AxA and SH cells. (**A**) Representative Western blot analysis of BRAF pathway proteins in AxA and SH shNS and shMTA1 cells. β-Actin served as the loading control. (**B**) Densitometric quantification of Western blot data demonstrate significant upregulation of BRAF wt in SH cells and significant downregulation of BRAF mut and pAKT/AKT following MTA1 knockdown. Statistical analysis was performed using *t*-test. Data are presented as mean ± SEM (*n* = 4–5). * *p* < 0.05; *** *p* < 0.001. #, not detected.

To investigate potential crosstalk between MTA1 and BRAF signaling, we examined the expression of wild-type (BRAF wt) and mutant BRAF V595E (BRAF mut) proteins, together with the downstream PI3K/AKT pathway, by assessing phosphorylated AKT (pAKT). As shown in Figure 4, BRAF wt was expressed in both cell lines, with substantially higher levels in SH cells, whereas BRAF mut was detected only in AxA cells. MTA1 silencing increased BRAF wt levels in SH cells. Importantly, BRAF mut expression was markedly reduced in AxA cells following MTA1 knockdown. Despite these differential effects on BRAF expression, pAKT levels were decreased in both AxA and SH cells after MTA1 silencing. The concomitant reduction in BRAF mut and pAKT in AxA cells suggests that MTA1 contributes to the maintenance of oncogenic BRAF signaling and its downstream PI3K/AKT pathway. These findings raise the possibility that targeting MTA1 may represent a therapeutic strategy to suppress BRAF-mutant canine UC, particularly in tumors that have acquired resistance to BRAF-targeted therapies.

**Figure 5:**
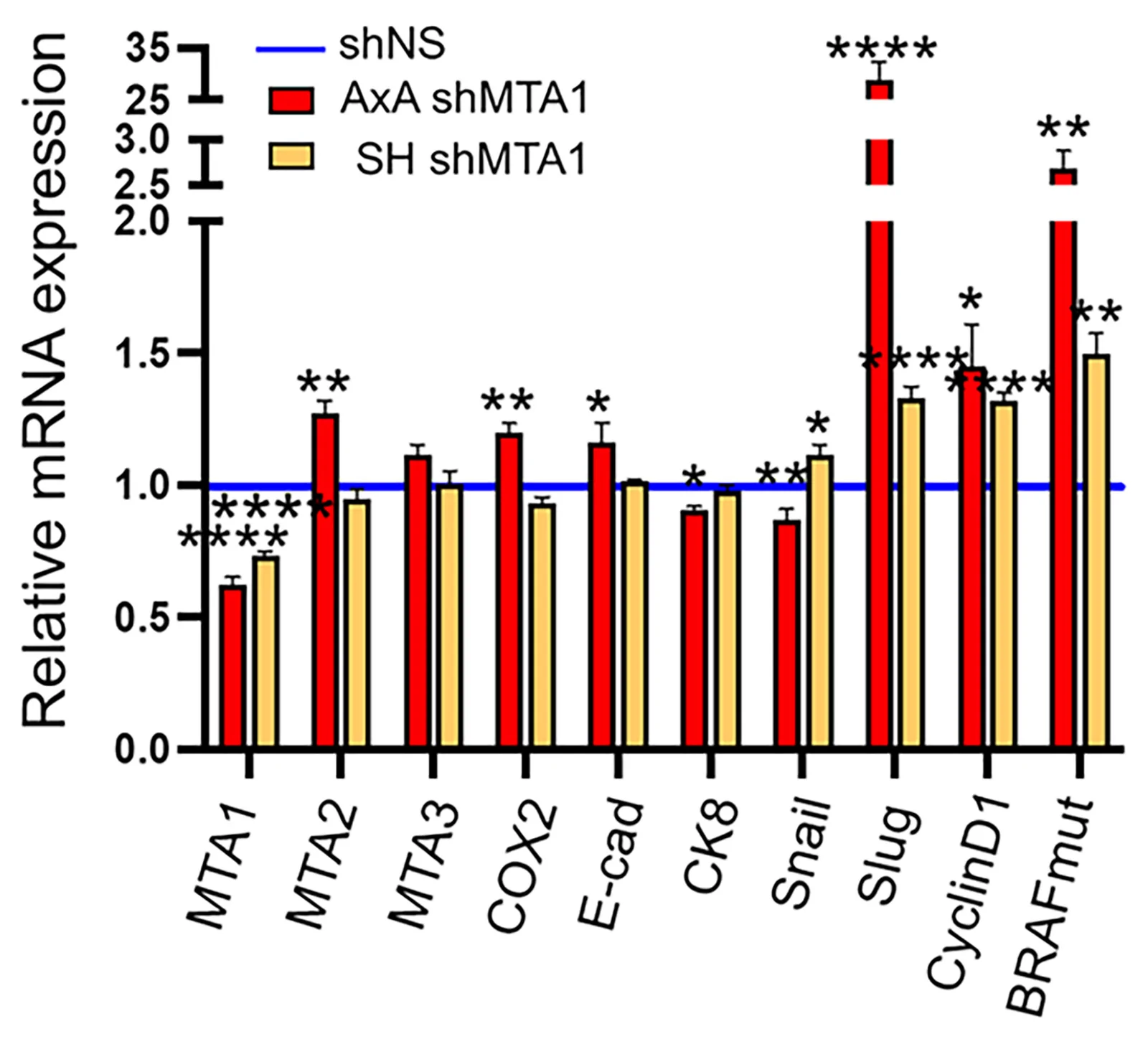
MTA1 knockdown differentially regulates EMT-associated genes and BRAF mut expression in canine UC AxA and SH cells. Representative qRT-PCR analysis of EMT-associated markers and BRAF mut in MTA1-expressing (shNS) and MTA1-knockdown (shMTA1) AxA and SH cells. qRT-PCR was performed in triplicate using three independent biological experiments. Relative mRNA expression was calculated using the 2^−ΔΔCt^ method. Statistical analysis was performed using unpaired two-sample *t*-tests for each individual gene, followed by Bonferroni correction for multiple comparisons. Data are presented as mean ± SEM (*n* = 3). * *p* < 0.05; ** *p* < 0.01; **** *p* ≤ 0.0001.

To identify whether the observed protein changes in MTA1 knockdown cells are regulated at the transcriptional level, we next examined the corresponding mRNA levels of the markers using qRT-PCR (Figure 5). Results showed that Cyclin D1 mRNA levels were correspondingly increased in both AxA and SH MTA1 knockdown cells. Snail and Slug mRNA expressions were similarly increased in SH MTA1 knockdown cells corresponding to their increased protein expression. However, in AxA MTA1 knockdown cells Snail mRNA levels were decreased in contrast to its significantly increased protein levels. A slight increase in E-cad mRNA levels was detected in AxA MTA1 knockdown cells, but not in SH knockdown cells. Surprisingly, while Slug protein levels were very low in AxA cells, Slug mRNA levels were extremely high suggesting that post-transcriptional or post-translational events play a role in the resultant undetectable levels of Slug protein in AxA. Another unexpected finding was the lack of concordance between BRAF mutant mRNA and protein expression. Although BRAF mutant transcripts were increased in both AxA-shMTA1 and SH-shMTA1 cells, BRAF mutant protein levels were reduced in AxA-shMTA1 cells and undetectable in SH cells. These results suggest that MTA1 silencing may trigger compensatory transcriptional responses while simultaneously impairing BRAF mutant protein translation or stability, highlighting a complex regulation of BRAF signaling by MTA1.

### 2.4. Effects of MTA1 on Xenograft Tumor Growth

**Figure 6:**
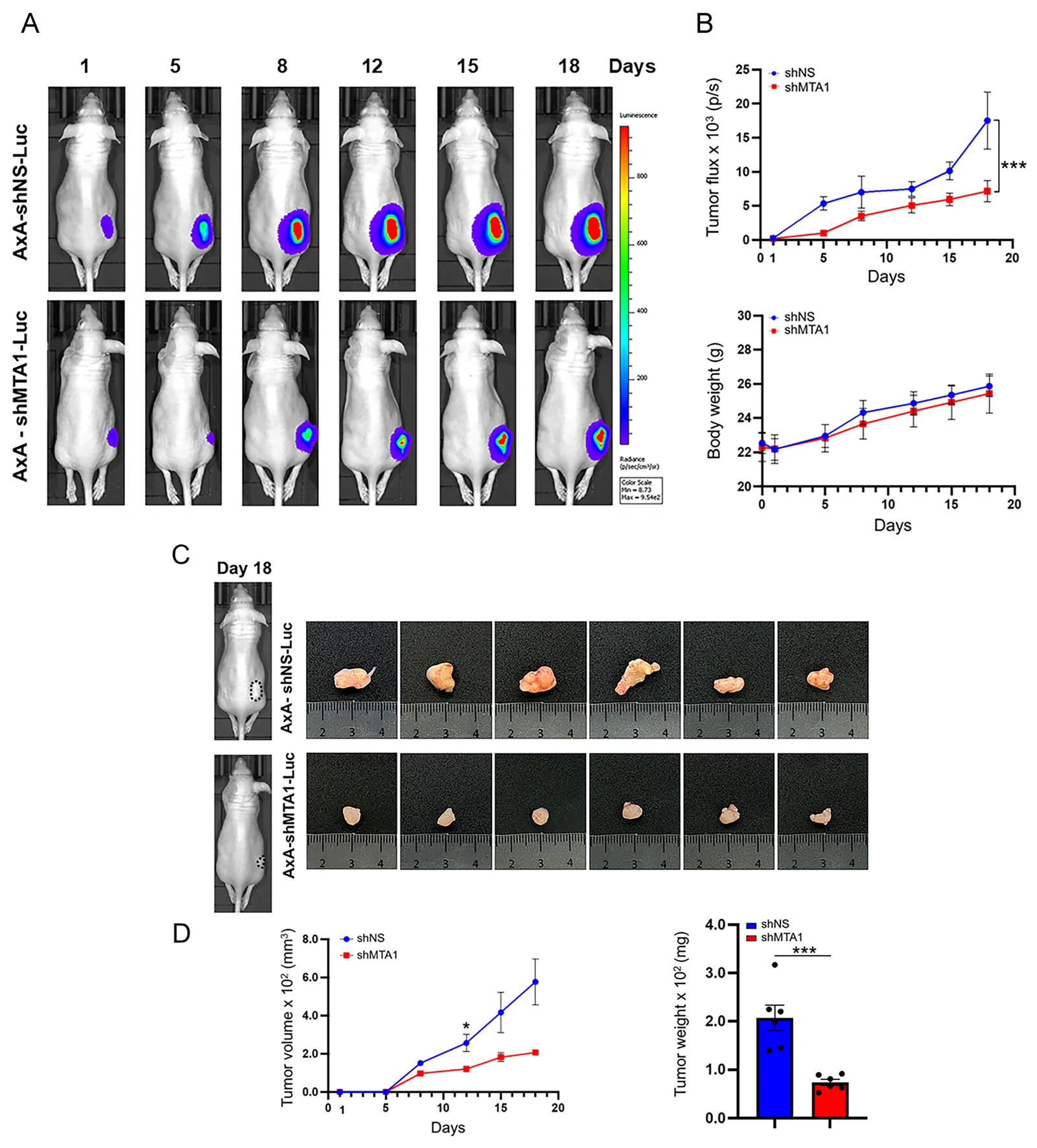
MTA1 silencing suppresses tumor growth in an AxA xenograft mouse model. (**A**) Representative bioluminescence (BL) images of mice bearing luciferase-tagged AxA-shNS and AxA-shMTA1 xenografts. Tumor growth was monitored by BL imaging twice weekly. (**B**) Quantitative analysis of tumor-associated BL (total flux, photons/s) over time (top). Data are presented as mean ± SEM from six mice per group. Bottom, average body weight of mice during the study, showing no significant differences between groups, indicating the absence of overt toxicity. (**C**) Representative images of mice bearing tumors at the study endpoint (left) and corresponding excised tumors (right) from each group (*n* = 6). (**D**) Tumor volume measured by digital calipers throughout the study (left), demonstrating significantly reduced tumor growth in the AxA-shMTA1 group beginning on day 12. Right, final tumor weights were significantly lower in the AxA-shMTA1 group than in controls. Statistical analysis was performed using two-way ANOVA. Data are presented as mean ± SEM. * *p* < 0.05; *** *p* < 0.001.

To investigate the role of MTA1 in canine UC growth in vivo, we employed a subcutaneous xenograft mouse model. AxA BRAF-mutant cells were selected because they exhibited the highest MTA1 expression and the most efficient MTA1 knockdown. Luciferase-tagged AxA-shNS and AxA-shMTA1 cells were validated for MTA1 and luciferase expression before implantation (Supplementary Figure S1), and tumors were allowed to establish for 18 days. Tumor growth was monitored twice weekly by bioluminescence (BL) imaging and digital caliper measurements. As shown in Figure 6A, control AxA-shNS tumors displayed rapid growth, whereas MTA1 knockdown markedly delayed tumor progression. Quantitative BL analysis revealed significantly lower tumor-associated light emission in the AxA-shMTA1 group on day 18 (Figure 6B, *top*). Consistent with these findings, MTA1 knockdown significantly reduced both final tumor volume and tumor weight compared with control xenografts (Figure 6C,D). No MTA1 knockdown-related adverse effects were observed, as evidenced by stable body weight (Figure 6B, *bottom*) and normal animal behavior throughout the study.

**Figure 7:**
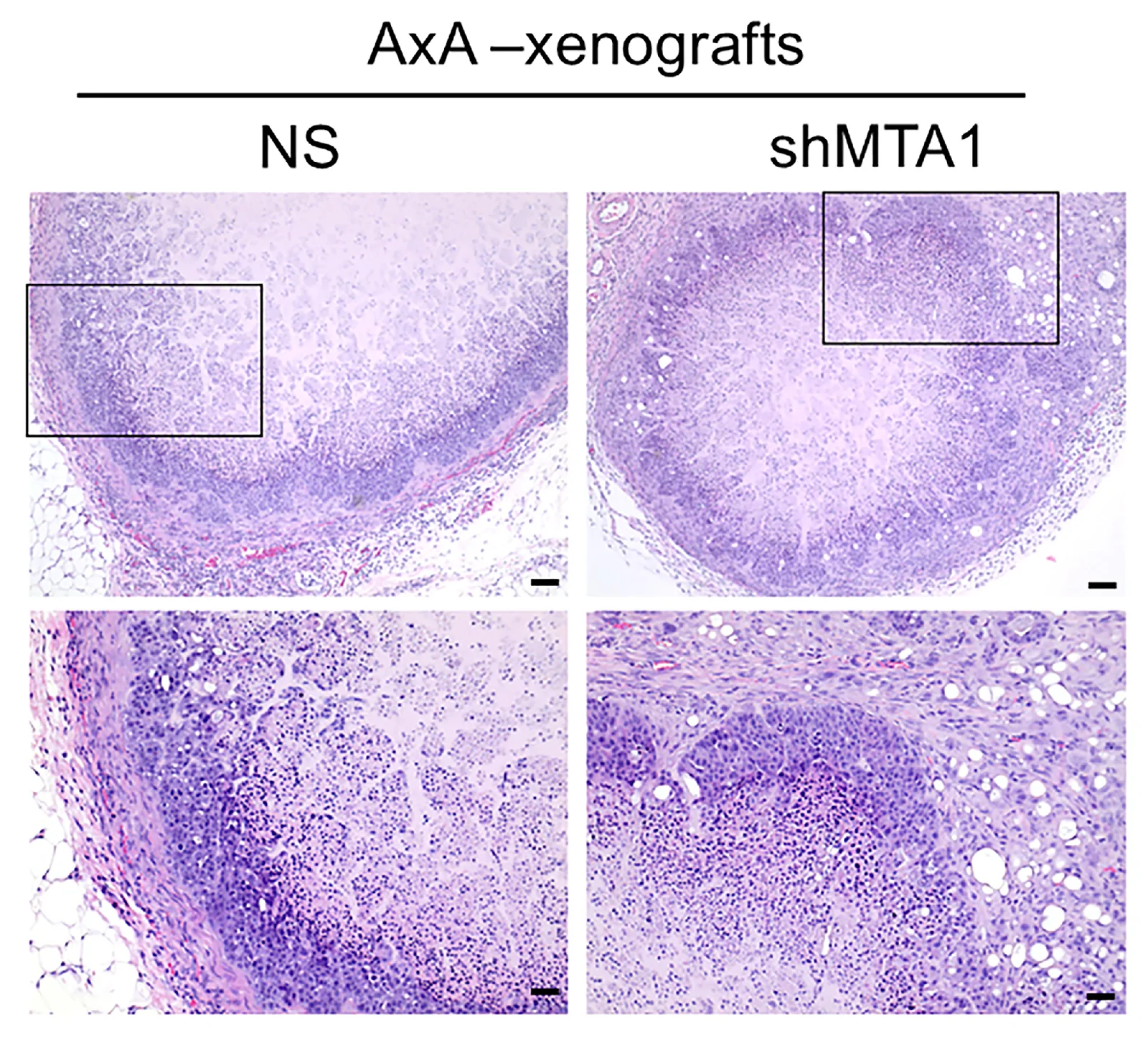
Histopathological analysis of AxA-shNS-Luc and AxA-shMTA1-Luc xenograft tumors. Representative hematoxylin and eosin (H&E)-stained sections of xenograft tumors from AxA-shNS-Luc and AxA-shMTA1-Luc mice. Histopathological examination revealed similar overall tumor architecture in both groups. Top: low-magnification images (scale bar = 100 µm). Bottom: high-magnification images (scale bar = 50 µm).

Histopathological evaluation of excised tumors from both control (AxA-shNS-Luc) and MTA1-silenced (AxA-shMTA1-Luc) groups revealed multinodular, densely cellular, unencapsulated neoplasms with extensive central necrosis, more pronounced in MTA1 knockdown tissues (Figure 7). Tumors consisted of polygonal neoplastic cells arranged in lobular and occasional papillary patterns supported by fibrous stroma. Cells exhibited marked nuclear and cellular pleomorphism, prominent nucleoli, abundant eosinophilic cytoplasm with occasional clear intracytoplasmic vacuoles, distinct cell borders, and frequent mitotic figures. The xenografts were surrounded by desmoplastic connective tissue with variable lymphocytic and neutrophilic infiltration at the tumor periphery.

**Figure 8:**
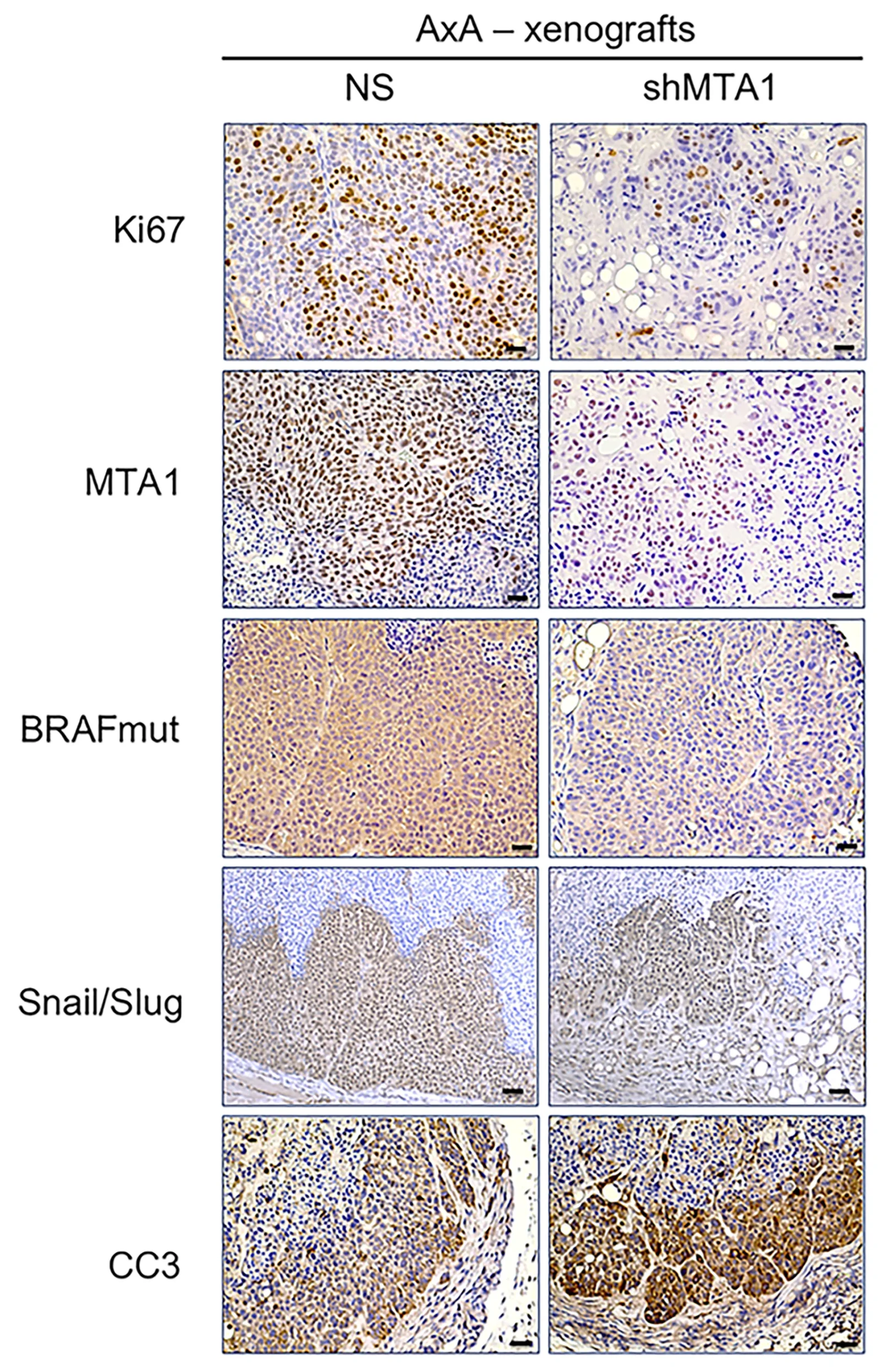
Immunohistochemical analysis of proliferation, BRAF signaling, EMT, and apoptosis markers in AxA xenograft tumors. Representative immunohistochemical (IHC) staining for Ki67, MTA1, BRAF mut, Snail/Slug, and cleaved caspase-3 (CC3) in tumor sections from AxA-shNS-Luc and AxA-shMTA1-Luc xenografts. Control (AxA-shNS-Luc) tumors exhibited strong immunoreactivity for Ki67, MTA1, BRAF mut, and Snail/Slug. In contrast, MTA1-knockdown tumors showed predominantly strong CC3 cytoplasmic staining versus weak staining in control tissues suggesting enhanced apoptotic cell death. Scale bar = 20 µm.

Figure 8 shows that consistent with reduced tumor growth, MTA1-silenced xenografts displayed lower proliferative activity, as evidenced by decreased Ki67 staining, and showed minimal MTA1 expression, confirming sustained target knockdown. Mutant BRAF expression was also markedly reduced in MTA1-silenced tumor compared with controls, supporting a role for MTA1 in regulating BRAF signaling in canine UC. Notably, Snail/Slug staining in MTA1-silenced tumors was predominantly nuclear, whereas control tumors exhibited primarily cytoplasmic localization, suggesting altered regulation of EMT-associated transcriptional activity following MTA1 suppression. Furthermore, MTA1-silenced tumors demonstrated strong cleaved caspase-3 staining, indicating increased apoptotic activity in vivo.

## 3. Discussion

In our previous study, we identified MTA1 as an overexpressed factor in canine UC, with elevated levels associated with adverse clinicopathological features and metastasis. These findings suggest that MTA1 may promote UC progression and metastatic spread through regulation of EMT [25]. In this study, we present the first integrated functional analysis of MTA1 in canine UC. By combining molecular profiling with in vitro and in vivo models, we demonstrate the consequences of MTA1 silencing on tumor cell growth, metastatic behavior, EMT-associated pathways, and BRAF signaling. Validation in xenograft models further establishes MTA1 as a key regulator of canine UC progression and supports its potential as a therapeutic target.

Studies have discovered that MTA family members participate in EMT events: both MTA1 and MTA2 support tumor progression, promote EMT and contribute to metastasis in various cancers [36,37,38,39,40,41,42,43]. MTA3 exhibits a context-dependent role in cancer, functioning either as a tumor suppressor, as indicated by its reduced expression in advanced tumors, or as an oncogene, where increased expression correlates with tumor progression and poor prognosis [44,45,46,47,48,49,50].

High MTA1 expression is inversely correlated with E-cadherin levels in multiple cancers, including human mesothelioma and non-small cell lung cancer, a relationship that we also observed in canine UC [25,51]. Snail and Slug are key drivers of EMT, promoting repression of E-cadherin and induction of mesenchymal markers such as vimentin. MTA1 and Snail/Slug exhibit a coordinated role in driving EMT and cancer progression, with both exhibiting similar expression patterns in invasive tumors and cancer cell lines [38,51]. Functionally, MTA1 cooperates with Snail to repress E-cadherin expression and promote EMT by directly targeting EMT-related genes, including E-cadherin [52,53]. While MTA1 and MTA2 drive EMT through E-cadherin repression, MTA3 opposes EMT by suppressing Snail-mediated E-cadherin downregulation [37,54]. In our study, MTA1 silencing decreased MTA2 and MTA3 expression and increased Snail protein levels in both AxA and SH canine UC cells; however, E-cadherin expression remained unchanged, suggesting the presence of compensatory mechanisms regulating EMT in these cells.

Both MTA1 and COX2 are frequently overexpressed in cancer and are associated with invasion, metastasis, and poor prognosis. Consistent with their pro-tumorigenic roles, a positive correlation between MTA1 and COX2 expressions has been reported in tumor tissues, including canine UC, while both proteins are inversely associated with E-cadherin expression [25,55]. Mechanistically, MTA1 has been shown to regulate COX2, as MTA1 overexpression increased COX2 levels, whereas MTA1 silencing reduced COX2 expression in cancer cells and xenograft tumors [56]. Similarly, in the present study, MTA1 knockdown resulted in a modest reduction in COX2 expression in both canine UC cell lines, further supporting a positive regulatory relationship between MTA1 and COX2.

The relationship between MTA1 and Cyclin D1 appears to be context-dependent. In gastric cancer cells, MTA1 positively regulates Cyclin D1 expression and promotes a malignant phenotype [57]. Similarly, MTA1 silencing in MDA-MB-231 breast cancer cells reduced Cyclin D1 levels and induced G0/G1 cell-cycle arrest, although this effect was not observed in MCF7 cells [58]. Consistent with a positive regulatory role, we previously demonstrated that MTA1 knockdown significantly decreased Cyclin D1 expression in human prostate cancer cells and xenograft tumors [28,59]. In contrast, the present study revealed that MTA1 silencing increased Cyclin D1 expression at both the mRNA and protein levels in canine AxA and SH UC cells, highlighting a cell type- and context-specific regulation of Cyclin D1 by MTA1.

Evidence linking MTA1 to CK8 expression is currently lacking, and the contribution of CK8 to EMT has not been fully elucidated [60]. EMT is a dynamic and often partial process, particularly at the invasive tumor front, where epithelial markers such as CK8 may be retained despite alterations in other EMT-associated proteins. Consistent with this complexity, vimentin was undetectable in both AxA and SH cells, indicating that these cells may undergo only a partial EMT. Moreover, MTA1 knockdown produced opposite effects on CK8 expression, increasing its levels in AxA cells while decreasing them in SH cells, suggesting that MTA1 regulates CK8 in a cell context-dependent manner during EMT.

One of the most significant findings of this study is the identification of a link between MTA1 and BRAF signaling in canine UC. The BRAF/MAPK pathway, driven by the BRAF V595E mutation, is a major oncogenic pathway in canine UC, particularly in Scottish Terriers, while COX2 and PI3K/AKT signaling also contribute to disease progression [10,11]. Despite the central role of BRAF signaling in canine UC, evidence connecting MTA1 to this pathway has been limited, although some reports have implicated MTA1 and other epigenetic regulators in resistance to BRAF- and EGFR-targeted therapies [61,62,63]. Our data demonstrate that MTA1 silencing reduced mutant BRAF expression in AxA cells and increased wild-type BRAF expression in SH cells, suggesting that MTA1 may influence BRAF signaling in a context-dependent manner. To further explore MTA1-associated signaling networks, we evaluated AKT activation and ultimately found that pAKT levels were decreased following MTA1 knockdown in AxA cells. This observation is consistent with our previous studies demonstrating a positive relationship between MTA1 and AKT signaling [26,28,64,65], and suggests that MTA1 may promote canine UC progression, at least in part, through coordinated regulation of the BRAF/MAPK and PI3K/AKT/mTOR pathways.

Finally, our in vivo studies demonstrated that MTA1 knockdown significantly suppressed AxA xenograft growth through reduced tumor cell proliferation and increased necrosis and apoptosis. Notably, mutant BRAF expression was also decreased in MTA1-silenced tumors, consistent with our in vitro findings, further supporting a functional link between MTA1 and BRAF signaling in canine UC. Despite the increased total Snail expression following MTA1 silencing, tumor growth and EMT-associated traits were diminished, suggesting that Snail activity may be regulated post-translationally. Because phosphorylation critically determines Snail stability and transcriptional activity, future studies examining Snail phosphorylation may provide important mechanistic insights into its relationship with MTA1 and its role in MTA1-mediated EMT in canine UC.

Regardless of adaptive molecular changes observed in vitro, MTA1 knockdown consistently resulted in significant inhibition of tumor growth in vivo, underscoring its dominant tumor-promoting role in the physiological tumor microenvironment. The discrepancy between in vitro signaling adaptations and in vivo tumor suppression likely reflects the greater complexity of in vivo regulatory constraints, including stromal interactions and microenvironmental limitations that may restrict compensatory pathway activity. Collectively, these data support a model in which MTA1 functions as a central regulator integrating BRAF/MAPK and PI3K/AKT signaling with EMT and proliferative networks. This positions MTA1 as a key molecular node in canine UC progression and a promising target for future interceptive and therapeutic strategies.

**Figure 9:**
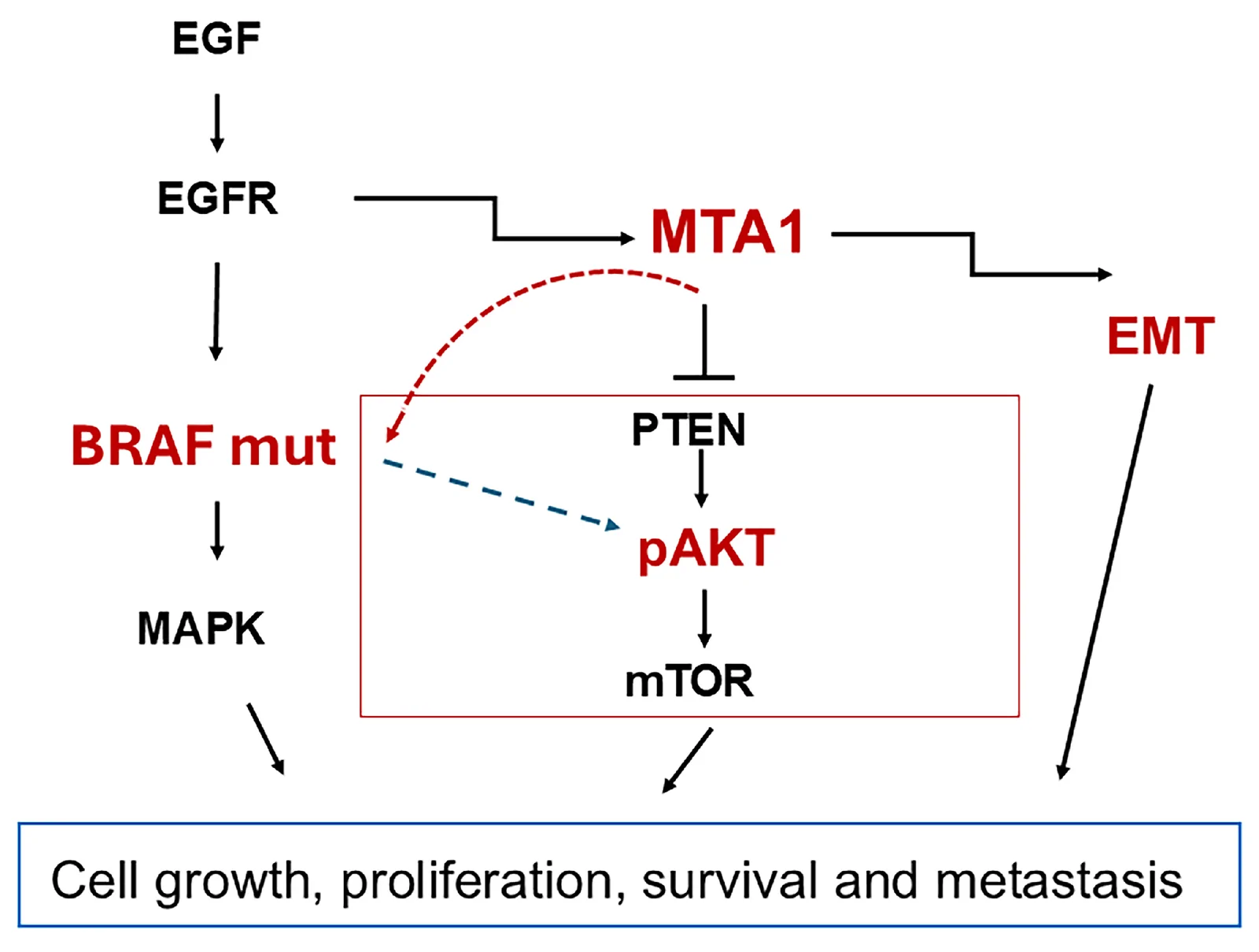
Proposed model of MTA1-mediated regulation of BRAF signaling in canine UC. MTA1 promotes BRAF V595E-driven canine UC progression by enhancing PI3K/AKT and EMT-associated signaling, thereby supporting tumor growth and malignant progression. In BRAF-mutant, drug-sensitive UC cells, MTA1 knockdown suppresses BRAF mut expression and AKT phosphorylation while inducing compensatory changes in EMT- and proliferation-associated markers. These observations identify MTA1 as a potential therapeutic target and provide a rationale for the future development of combination therapies targeting both MTA1 and BRAF to enhance treatment efficacy and delay or overcome acquired resistance in BRAF-mutant canine UC.

Based on our findings, we propose that MTA1 functions as an upstream regulator of BRAF-driven signaling in canine UC (Figure 9).

MTA1 overexpression may sustain BRAF-mutant expression and downstream oncogenic pathways, including the PI3K/AKT survival axis, thereby promoting tumor progression and therapeutic resistance. Conversely, MTA1 silencing reduced BRAF-mutant expression both in vitro and in vivo and was accompanied by decreased AKT activation, suggesting that MTA1 contributes to the maintenance of these signaling networks. These findings suggest that pharmacological inhibition of MTA1 could enhance the efficacy of BRAF-targeted therapies and potentially delay or overcome the development of therapeutic resistance. Therefore, combined targeting of MTA1 and BRAF signaling warrants further investigation as a therapeutic strategy for canine UC.

## 4. Materials and Methods

### 4.1. Cell Culture

The canine invasive UC cell lines K9TCC-PU-Sh (SH) and K9TCC-PU-AxA (AxA) were a generous gift from Dr. Knapp, Purdue University, IN [25,35]. Cells were cultured in DMEM/F12 containing 10% fetal bovine serum (FBS) and maintained at 37 °C with 5% CO_2._ Cells were regularly tested for mycoplasma using Universal Mycoplasma Detection Kit (ATCC, Manassas, VA, USA) and found to be mycoplasma-free.

### 4.2. Establishment of MTA1 Knockdown Cells

MTA1 knockdown in AxA and SH cells was achieved using shRNA lentiviral constructs via a technique described previously [33,34], and the clones with the most efficient knockdown were chosen for current study. Briefly, canine UC cells were transduced with the GIPZ MTA1 lentiviral shRNAs and a GIPZ Non-silencing (NS) lentiviral shRNA (GE Heathcare Dharmacon, Lafayette, CO, USA). These constructs contain a puromycin-resistant gene for selection of transduced cells and TurboGFP for monitoring the selection under the fluorescence microscope. Viral particles were obtained using pCMV-ΔR8.91 packaging plasmid and pMD.G envelope plasmid (Addgene, Cambridge, MA, USA) introduced into HEK293T cells. AxA and SH cells were infected using culture media with lentiviral particles and 4 μg/mL polybrene transfection enhancer (Sigma-Aldrich, St. Louis, MO, USA). On day 3 post-transduction, selection was initiated with 1 μg/mL puromycin (Sigma-Aldrich, St. Louis, MO, USA), GFP-positive clones were selected under the microscope and further propagated in the presence of puromycin. The best of three shRNA clones showing the most efficient knockdown was selected for the current study.

### 4.3. Proliferation Assay

The cell proliferation assay was performed for 10 days for AxA and SH clones (shNS and shMTA1); 1 × 10^4^ cells were seeded in 60 mm tissue culture dishes and culture media was replenished every two days. Cells were counted using Trypan Blue staining every alternate day for a total period of 10 days. Cells were seeded in triplicate, and experiments were repeated twice for each cell line. Population doubling time was determined using the following equation:

$$\frac{T\left(ln2\right)}{\ln\left(\frac{Xe}{Xb}\right)},$$

where *T* = time, *Xe* = number of cells at the end of log growth phase, and *Xb* = number of cells at the beginning of log growth phase.

For AxA cells, log growth phase was determined to be between days 4–10, and for SH cells, log growth phase was determined to be between days 6–10.

### 4.4. Colony Formation Assay

AxA and SH clones (shNS and shMTA1) were seeded at a density of 2 × 10^3^ cells/well into a 6-well culture plates and incubated at 37 °C for 14 days. Serum-deprived media (1% FBS) was refreshed every other day. When colonies were visible (i.e., containing > 50 cells), cells were fixed and stained with 0.01% crystal violet for 30 min. Images were taken using a ChemiDoc Imaging System (Bio-Rad, Hercules, CA, USA) and colonies were counted using ImageJ software v1.54t (NIH, Bethesda, MD, USA). Experiments were performed in triplicates and repeated three times.

### 4.5. Wound Healing Assay

AxA and SH clones (shNS and shMTA1) were seeded into a 6-well culture plate and incubated in culture media until they reached ~90% confluence. Wounds (3/well) were scratched through the cell monolayer using a sterile 200 µL pipette tip and debris removed by washing with PBS. The cells were then starved of serum (0.1% for AxA and 0.5% for SH) for the next 36 h. Wound images were taken using EVOS FL Core microscope (Thermo Fisher Scientific, Waltham, MA, USA) and wound areas were analyzed using Image J software (NIH, Bethesda, MD, USA). Wound closure (%) was determined using the following formula: $\left(\frac{W_{t=0}-W_{t=x}}{W_{t=0}}\right)\times 100$, where W = wound area. Experiments were conducted in triplicates and repeated three times.

### 4.6. Western Blot Analysis

Western blot analysis was carried out as previously described [26]. Briefly, total protein was isolated from MTA1-expressing and MTA1 knockdown cells. A total of 30 µg of lysate was separated in 10–15% polyacrylamide gels, transferred onto polyvinylidene difluoride (PVDF) membranes, blocked with 5% milk, and then probed with primary antibodies listed in Supplementary Table S1. β-actin was used as a loading control. Signals were developed using enhanced chemiluminescence and detected by ChemiDoc Image Lab v 3.0.1. (Bio-Rad, Hercules, CA, USA). Band intensities were measured using Image J (NIH, Bethesda, MD, USA).

### 4.7. Real-Time qPCR

RNA was isolated using a RNeasy Mini Kit (Qiagen, Hilden, Germany) and reverse transcribed to cDNA using SuperScript III Reverse Transcriptase (Thermo Fisher Scientific, Somerset, NJ, USA). qRT-PCR was performed on the Applied Biosystems QuantStudio 5 Real-Time PCR System (Thermo Fisher Scientific, Somerset, NJ, USA). Canine-specific primers are listed in Supplementary Table S2. β-actin was used for normalization. Fold changes in mRNA expression were estimated by the double-delta C_t_ (2^−ΔΔCt^) method [66].

### 4.8. Generation of Stable Cells Tagged with Luciferase and Bioluminescent Imaging

We transduced AxA-shNS and AxA-shMTA1 cells with the luciferase (Luc) construct (a gift from Dr. R. Graeser, Dept of Medical Oncology, Freiburg, Germany) and selected stable clones using G418 selection (Caymen Chemicals, Ann Arbor, MI, USA). Cells were expanded and tested in vitro for their Luc activity. Briefly, cells tagged with Luc were serially diluted in a flat, clear bottom 96-well plate (Costar, Corning, NY, USA). D-luciferin (150 µg/mL) was added to each well, and the plate was incubated at 37 °C, 5% CO_2_ for 10 min after which images were taken using IVIS Lumina LT III (Revvity, Hopkinton, MA, USA) (Supplementary Figure S1).

### 4.9. Mouse Xenograft Tumor Model

Four- to five-week-old female nude mice (Hsd:Athymic Nude-Foxn1nu) were purchased from Envigo (Inotiv, Indianapolis, IN, USA) and housed under pathogen-free conditions and allowed to acclimatize. Animals were randomly assigned to two groups: AxA-shNS-Luc (control) or AxA-shMTA1-Luc cells (*n* = 6/group). A cell suspension containing 2 × 10^6^ cells in 100 µL Matrigel/PBS (1:1)) was injected s.c. into right posterior flank. In vivo BL images were taken twice weekly. Briefly, mice were anesthetized using isoflurane delivered by an XGI-8 Gas Anesthesia System (Caliper Life Sciences, Hopkinton, MA, USA). They were then injected intraperitoneally (i.p.) with 150 mg/kg bw D-luciferin, weighed, and measured for tumor volume (mm^3^) using digital calipers according to the following formula: $\frac{L\;\times \;W^{2}}{2}$, where L = longest diameter, and W = diameter perpendicular to L. Mice were transferred to the IVIS where they continued to receive 2% isoflurane via a mouse manifold. BL images were acquired 20 min post D-luciferin injection using exposures between 2–10 s, F-stop f/8, and medium binning. Grayscale images were captured using auto-exposure. BL images were normalized (loaded as a group) and radiance or total flux (photons/sec) from regions of interest were measured. Tumors grew for 18 days, after which mice were sacrificed. At necropsy, s.c. tumors were excised, weighed, and fixed in 10% neutral-buffered formalin for paraffin embedding and sectioning (IDEXX BioAnalytics, Columbia, MO, USA).

All animal experimental procedures were carried out according to protocols approved by the IACUC committee of the Long Island University, in accordance with the National Institutes of Health Guide for the Care and Use of Laboratory Animals.

### 4.10. Histopathology and Immunohistochemistry

Formalin-fixed, paraffin-embedded sections were stained with hematoxylin and eosin (H&E) for histopathologic evaluation. Immunohistochemistry was applied to evaluate Ki67, MTA1, BRAF, Snail/Slug, and cleaved caspase 3 (CC3) expression in xenograft tissues as previously described [25]. Briefly, 5 µm sections from formalin-fixed paraffin-embedded blocks were deparaffinized and hydrated. Antigen retrieval was performed using Antigen Unmasking Solution (Vector Labs, CA) and steam. Endogenous peroxidase activity was quenched using 3% H_2_O_2_. Tissues were blocked, incubated overnight at 4 °C with the primary antibodies (Supplementary Table S1). Respective biotinylated secondary antibodies were used from the Vectastain ABC Elite kit, followed by a 30 min incubation in ABC reagent. Staining was developed using the ImmPACT DAB kit (Vector Labs, Newark, CA, USA) and nuclear counterstaining was performed using Gill’s hematoxylin III (1:10). Tissues were then dehydrated, cleared, and mounted. Images were viewed and recorded on an EVOS XL Core Microscope (Thermo Fisher Scientific, Somerset, NJ, USA).

### 4.11. Statistical Analysis

All data represent at least three independent experiments. The statistical differences between groups were determined by a one-way ANOVA, mixed-effects model, or *t*-test. All tests were performed using GraphPad Prism software v 11.0.2 (GraphPad Software, La Jolla, CA, USA). *p*-values < 0.05 were considered statistically significant.

## 5. Conclusions

In conclusion, this study demonstrates that MTA1 plays a critical role in regulating canine UC progression while also revealing a complex and context-dependent cellular response to MTA1 knockdown. In vitro, MTA1 silencing resulted in a paradoxical increase in EMT- and proliferation-associated markers, including Snail/Slug and Cyclin D1, suggesting activation of compensatory molecular programs. In parallel, MTA1 knockdown reduced mutant BRAF expression and was accompanied by decreased AKT phosphorylation, indicating suppression of oncogenic BRAF signaling and its downstream PI3K/AKT pathway. Collectively, these findings suggest that MTA1 inhibition may represent a promising therapeutic strategy for BRAF-mutant canine UC and may have particular value in combination with BRAF-targeted therapies to delay or overcome acquired drug resistance. Further studies using BRAF inhibitor-resistant models are warranted to validate this therapeutic approach.

## Data Availability

The data presented in this study are available on request from the corresponding author but are not publicly accessible.

## Supplementary Materials

The following supporting information can be downloaded at: https://www.mdpi.com/article/10.3390/ijms27167272/s1.

## Abbreviations

AKT	V-akt murine thymoma viral oncogene (protein kinase B)
ANOVA	Analysis of variance
ATCC	American Type Culture Collection
AxA	K9TCC-PU-AxA cell line
BL	Bioluminescence
BRAF	v-Raf murine sarcoma viral oncogene homolog B
CC3	Cleaved caspase 3
CK8	Cytokeratin 8
COX2	Cyclooxygenase 2
DMEM/F12	Dulbecco’s Modified Eagle Medium/Nutrient Mixture F-12
E-cad	E-cadherin
EGF	Epidermal growth factor
EGFR	Epidermal growth factor receptor
EMT	Epithelial-to-mesenchymal transition
FBS	Fetal bovine serum
H&E	Hematoxylin and eosin
IHC	Immunohistochemistry
K9TCC	Canine transitional cell carcinoma cell line
Ki67	Cellular protein marker of proliferation
MAPK	Mitogen-activated protein kinase
MAPK/ERK	Mitogen-activated protein kinase/extracellular signal-regulated kinase
MTA1	Metastasis-associated protein 1
mTOR	Mammalian target of rapamycin
mut	Mutant
NS	Non-significant
NSAIDs	Nonsteroidal anti-inflammatory drugs
NuRD	Nucleosome remodeling and deacetylation complex
pAKT	Phosphorylated AKT
PI3K	Phosphoinositide 3-kinase
PTEN	Phosphatase and tensin homolog
PVDF	Polyvinylidene difluoride
SD	Standard deviation
SEM	Standard error of mean
shMTA1	MTA1 shRNA knockdown (MTA1-silenced)
shNS	Nonspecific shRNA (control)
Slug	Zing finger protein SNAI2
Snail	Zing finger protein SNAI1
SH	K9TCC-PU-Sh cell line
TCC	Transitional cell carcinoma
UC	Urothelial carcinoma
wt	wild-type
