# Supplementary material for: MTA1 Regulates EMT and BRAF Signaling Networks in Canine Urothelial Carcinoma
Source: Int J Mol Sci. 2026 Aug 14;27(16):7272. doi: 10.3390/ijms27167272 (PMC13513100; doi:10.3390/ijms27167272)
Supplement: Supplementary file 1 [file ijms-27-07272-s001.zip › ijms-4447612-supplementary.pdf]

Supplementary Material:

MTA1 Regulates EMT and BRAF Signaling Networks in Canine Urothelial Carcinoma

Gisella Campanelli, Nema Parkhomovsky , Chun Kuen Mak, Ching Yang and Anait S. Levenson

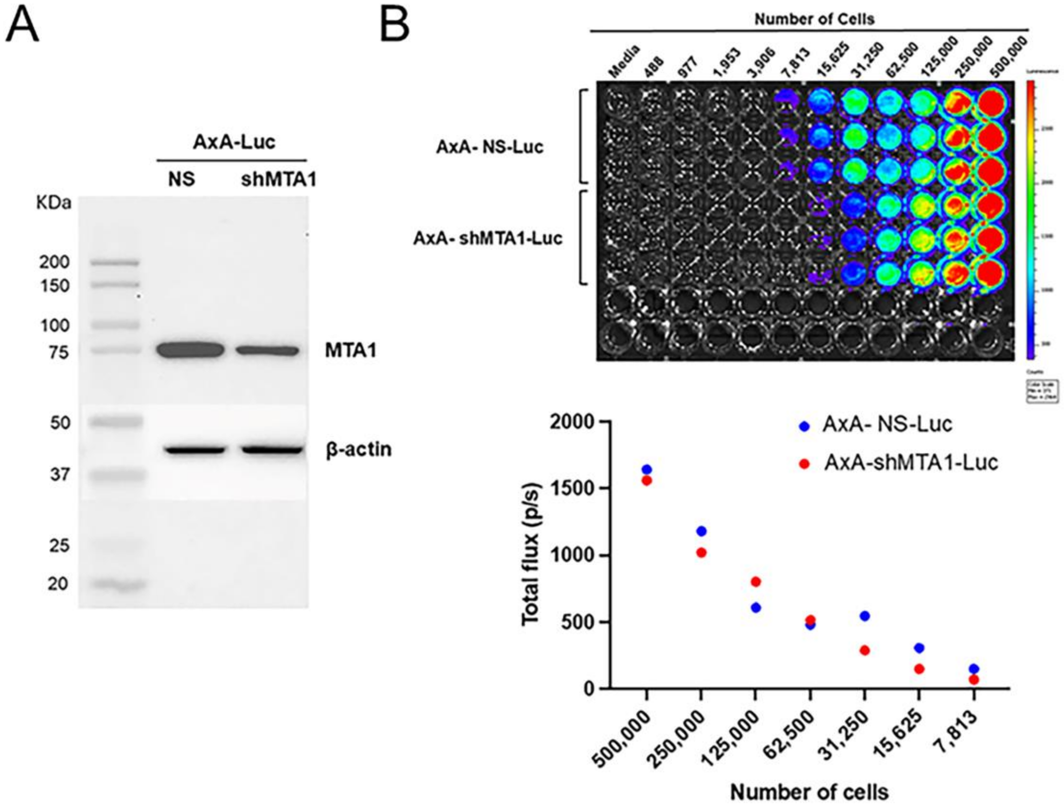

Figure S1. **(A)** Validation of MTA1 knockdown of AxA-NS and AxA-shMTA1 cells tagged with luciferase (Luc) prior to injection into mice. **(B**) Validation of specificity and sensitivity of Luc expression in AxA-NS-Luc and AxA-shMTA1-Luc cells in vitro. Cells showed reliable and reproducible luciferase activity with a radiance that positively correlated to the number of cells seeded. The subtle differences in radiance were not significant (*bottom*). These cells were used for the establishment of tumor xenografts.

Table S1. Antibodies used for Western Blot and IHC

Antibody	Application	Dilution	Source	Catalog#
β-actin	WB	1:5000	Santa Cruz Biotechnology	sc-69879
Akt	WB	1:1000	Cell Signaling	4691
BRAF	WB	1:100	Santa Cruz Biotechnology	sc-55522
BRAF V600E mutation	WB	1:1000	Abcam	ab228461
Cleaved Caspase 3	IHC	1:100	Cell Signaling Technologies	9664
COX2	WB	1:2000	Cayman Chemical Company	160107
Cyclin D1	WB	1:1000	Cell Signaling Technologies	2922
E-cadherin	WB	1:1000	Cell Signaling Technologies	3195
Ki67	IHC	Ready-to-Use	Dako – Agilent Technologies	GA626
MTA1	WB<br>IHC	1:1000<br>1:50	Cell Signaling Technologies	5647
MTA2	WB	1:100	Santa Cruz Biotechnology	sc-55566
MTA3	WB	1:100	Santa Cruz Biotechnology	sc-81325
p-Akt	WB	1:400	Cell Signaling Technologies	4060
Slug	WB	1:250	Cell Signaling Technologies	9585
Snail	WB<br>IHC	1:500<br>1:100	Cell Signaling Technologies<br>Proteintech	3879<br>13099-1-AP
Snail/Slug	IHC	1μg/ml	Abcam	Ab85936

Table S2. Primers used for qRT-PCR

**Canine Primer**	**Sequence**
β-actin forward	5′- CTT CCA GCC TTC CTT CCT GG -3′
β-actin reverse	5′- CCA GGG TAC ATG GTG GTT CC -3′
BRAF V595E-mut forward	5′- ATT TCA AGC CCC CAA AAT CT -3′
BRAF V595E-mut reverse	5′- GTA GCA CCT CAG GGT CCA AA -3′
CK8 forward	5′- AGC AGA AAA CCT CTC GGA GC -3′
CK8 reverse	5′- AGG CGG GAC TCC AGT TCT AT -3′
COX2 forward	5′- GCC CTA TAC ATC ATT CGA AGA AC -3′
COX2 reverse	5′- CAC CAT AAA GGG CCT CCA A -3′
Cyclin D1 forward	5′- CGG TAC ACC CCA CTT TCA CA -3′
Cyclin D1 reverse	5′- TCC AAG GAA AGA GGA GCA CC -3′
E-cadherin forward	5′- CAG CAT GGA CTC AGA AGA CAG AAG -3′
E-cadherin reverse	5′- TTC GGG GCA GCT GAT AGG -3′
MTA1 forward	5′- CGT GCA ACA GAA ACG CTT GA -3′
MTA1 reverse	5′- AAG ACT GGG CGG TGT AAC AG -3′
MTA2 forward	5’- GGC GCA GGG ACA TTT CTA GT -3’
MTA2 reverse	5’- AGA AGT GTC TTC TGC ACG GG -3’
MTA3 forward	5′- AAC AGC ATT TCT GGA ATG TCT GC -3′
MTA3 reverse	5′- TAT CAG GGG AAA GTG CAG TGT TG -3′
Slug forward	5′- AGT GAT TAT TTC CCC ATA TCT CTA TGA -3′
Slug reverse	5′- GTA GTC TTT CCT CTT CAT CAC TAA TGG -3′
Snail forward	5′- CCC AAG CCC AGC CGA TGA G -3′
Snail reverse	5′- CTT GGC CAC GGA GAG CCC -3′
